# Automated Fluid Management for Treatment of Rhabdomyolysis

**DOI:** 10.1155/2016/2932593

**Published:** 2016-11-24

**Authors:** Christian M. Beilstein, John R. Prowle, Christopher J. Kirwan

**Affiliations:** Adult Critical Care Unit, The Royal London Hospital, Barts Health NHS Trust, Whitechapel Road, London E1 1BB, UK

## Abstract

*Purpose*. Fluid therapy aimed at increasing urine output is a commonly employed strategy to prevent acute kidney injury (AKI) in critically ill patients with rhabdomyolysis. Automated fluid management has the potential to optimise urine output while avoiding fluid accumulation in rhabdomyolysis patients.* Methods*. In a single centre clinical service evaluation we compared a convenience sample of critically ill adults with rhabdomyolysis treated with automated fluid management using the RenalGuard® device to patients managed with manual fluid adjustment following our standard rhabdomyolysis protocol. Primary outcome was number of hours with urine output >2 mL/kg during first 48 h of therapy.* Results*. Eight patients treated with RenalGuard were compared to 28 patients treated with manual fluid management. Number of hours of target urine output was greater in the RenalGuard versus the Standard group (176/312 (56.4%) versus 534/1305 (40.9%); *p* < 0.01). Urine output was significantly higher in the first 24 h in the RenalGuard group (median (IQR) 4033 mL (3682–7363) versus 2913 mL (2263–4188 mL); *p* < 0.01). Fluid balance, electrolyte, diuretics, and bicarbonate use were comparable between groups.* Conclusions*. Automated fluid management resulted in a higher urine output more quickly in the treatment of rhabdomyolysis. Further research is needed to analyse the effect of diuresis-matched hydration for the prevention of AKI in rhabdomyolysis.

## 1. Introduction

Rhabdomyolysis is the dissolution of striped muscle and has numerous causes. The leakage of muscle-cell contents including electrolytes, myoglobin, and Creatine Phosphokinase (CK) into the blood stream has toxic effects on the kidneys in a number of ways and may lead to acute kidney injury (AKI). Renal injury is understood to be caused by intrarenal vasoconstriction, direct tubular toxicity, and tubular obstruction by Tamm-Horsfall protein containing casts all of which are precipitated by the presence of myoglobin. AKI is reported to complicate between 13 and 50% of cases of rhabdomyolysis [1]. Whilst peak myoglobin predicts AKI better, serum CK remains elevated for longer following rhabdomyolysis and is therefore more widely used to guide therapy [2, 3]. A CK level of greater 5000 U/l is widely accepted as threshold indicating serious muscle injury [4, 5].

The prevention of AKI in patients with significant rhabdomyolysis includes treating the underlying cause (e.g., fasciotomy to relieve compartment syndrome), fluid resuscitation (hypovolaemia is common at diagnosis), and maintaining high urine output with alkalinisation of the urine to prevent precipitation of casts in the renal tubules [6–8]. High volume haemofiltration or super high flux haemodialysis has been used for extracorporeal elimination of myoglobin in severe cases [9, 10].

Rhabdomyolysis is common in our critical care unit (about 7% of all admissions [11]) and treatment is a protocolled high fluid output/input strategy for any patient with a CK greater than 5000 U/l. The protocol targets a urine output of greater than 2 mL per kg estimated body weight (EBW) per hour, a urine pH of greater than 6, and allows the use of loop diuretics and intravenous sodium bicarbonate 1.26% to achieve this. The aim is to replace 100% urine output of the previous hour in the following hour. Our concern is that the delay in manual fluid replacement results in episodic hypovolaemia as it is always “behind” the previous hours output.

The RenalGuard System (PLC Medical Systems, Milford, Massachusetts, USA) works by replacing the patient's urine output millilitre-for-millilitre, minute-by-minute. This accurate, automated real-time replacement reduces the risk of over- or underhydration (i.e., episodic hypovolaemia) relative to standard infusion in the presence of desired high volume diuresis. RenalGuard has previously been tested and evaluated in patients at risk for developing acute kidney injury following coronary or peripheral angiography. It is believed that maintaining a high urine flow rate leads to lower concentration and a faster transit of potentially toxic molecules trough the kidneys, respectively, [12]. There are promising results which suggest a 50–80% relative risk reduction for the development of contrast-induced nephropathy [13, 14].

The aim of this clinical service evaluation is to demonstrate that the RenalGuard device can be safely and practically incorporated into a rhabdomyolysis treatment protocol for critically ill patients that will perform better than the established standard protocol. Our primary outcome measure was number of hours of urine output greater than 2 mL/kg/h. Secondary outcomes were primarily focused on safety (in terms of fluid balance and electrolyte disturbance) and usability, achieving daily set fluid balance, use of electrolyte substitution, loop diuretics and sodium bicarbonate, and premature protocol cessation.

## 2. Methods

### 2.1. Governance

This was a prospective clinical service evaluation audit of a commercial medical device and was registered as an audit with the Clinical Effectiveness Unit, Barts Health NHS Trust. All patients were managed using our existing clinical protocol for fluid therapy in rhabdomyolysis.

### 2.2. Setting

This single-center study was conducted in a 44-bedded general adult critical care unit in East London, United Kingdom. The case mix is split between medical (40%), surgical (30%), and major trauma (30%) patients.

### 2.3. Outcome Measures

Primary outcome measure: number of hours with urine output greater 2 mL/kg estimated body weight within the first 48 hours using a “standard” rhabdomyolysis protocol versus one incorporating the RenalGuard. Secondary outcome measures were the maintenance of a set fluid balance, the need for electrolyte replacement, the administration of loop diuretics and bicarbonate, and premature protocol cessation.

### 2.4. Patient Groups

Patients already on or with acute indications for renal replacement therapy or patients with a diagnosis of diabetes insipidus were excluded from use of out rhabdomyolysis management protocol. A convenience sample of patients from 01.01.2015 to 31.04.2015 with a CK greater than 5000 U/l were commenced on rhabdomyolysis treatment using the RenalGuard device if the RebalGuard was not in use already and also if there was a member of the investigating team present to support setting it up. The final decision regarding management of rhabdomyolysis protocol was with the treating clinician. Data from patients with a transient CK rise of <48 hours were prospectively excluded from our analysis as they would not receive 48 h of protocolled therapy. We compared automated fluid management with the RenalGuard to manual fluid management using the same therapeutic protocol. For our comparison group we audited charts from ICU patients with more than one CK measurement of >5000 U/l in our electronic clinical records system between 01.01.2014 and 31.12.2014. Patients were excluded if they required RRT, had a CK > 5000 U/I for less than 48 hours, and the treatment protocol stopped before 48 hours of treatment had occurred at the instruction of the treating clinician.

### 2.5. Protocol Application

Rhabdomyolysis protocol using manual fluid management was initiated and implemented by the clinical team without any external intervention and discontinued when the CK fell below 5000 U/l.

The RenalGuard protocol required the investigators to help set up and use the RenalGuard device for the bedside nurse who then followed the protocol independently. The use of the RenalGuard was discontinued by one of the following scenarios: CK fell below 5000 U/l, at the treating clinician's discretion or after 72 h of therapy. If required, the rhabdomyolysis protocol using manual fluid management was continued thereafter.

### 2.6. Observation Period

We collected observations on all patients for the first 48 hours after the first CK measurement of >5000 U/l.

### 2.7. Data Collection

All data was collected from the bedside charts, medical notes, and electronic records, retrospectively for the standard group and prospectively for the RenalGuard group. Baseline data included demographic information, diagnosis, and serum Creatinine value on admission to the critical care unit. At 12 h intervals following the diagnosis of rhabdomyolysis, total fluid input, output, and balance were recorded along with hourly urine output, electrolyte, and metabolic indicators from blood gas analysis. In both groups, hours off the ward (e.g., in radiology) were not counted to allow correct calculation of the primary outcome measure as the protocol was paused during these times. We also collected the dose of intravenous electrolyte substitution, loop diuretic, and sodium bicarbonate 1.26%. In addition we recorded >1 L deviations from the set fluid balance, new onset pulmonary oedema, and premature protocol cessation.

### 2.8. Data Analysis

The number of hours where the target urine output was >2 mL/kg was calculated for every 12-hour period. Average urine output (absolute and per kg) was calculated for every 24-hour period, fluid balance as well as deviation from a set target balance for the whole observation period (48 hours). Data is presented as median (interquartile range) for continuous variables and absolute or relative frequencies as percentages for categorical variables. Serial measures were analysed comparing the area under the curve using the trapezoid method as described by Matthews et al. [15]. Differences in between groups were compared using Mann–Whitney* U* test and Pearson's chi-square (*χ* ^2^) test with continuous and categorical date, respectively. All statistical analysis was carried out using IBM SPSS Statistics 22 (IBM Corp, USA) and Microsoft Excel 2011 (Microsoft Corp, USA).

## 3. Results

### 3.1. Baseline Characteristics

**Figure 1:**
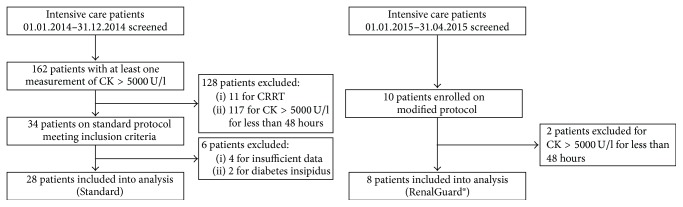
Recruitment flow chart.

We compared 8 patients using automated fluid management with RenalGuard to 28 patients who received manual rhabdomyolysis treatment (Figure 1). Baseline characteristics were comparable (Table 1).

### 3.2. Hours within Urine Output Target

**Figure 2:**
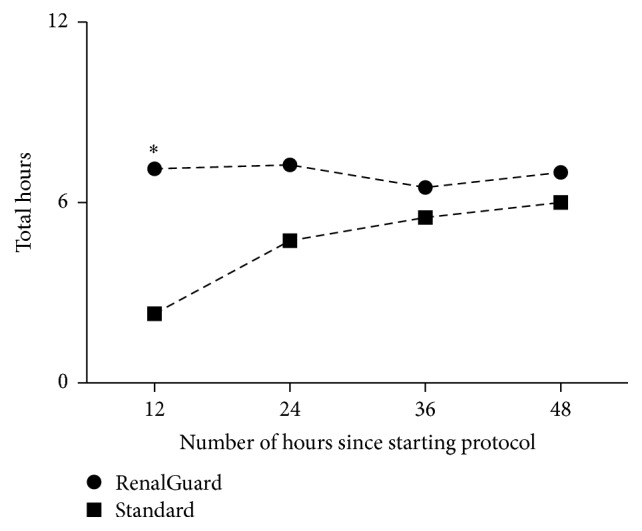
The number of hours of urine output >2 mL/kg in each 12 hours of treatment (^*∗*^ *p* < 0.01).

The urine output target of 2 mL/kg EBW/hour was reached in the first 48 hours in 56.4% hours (176 of 312) in the RenalGuard group and 40.9% (534 of 1305) in the Standard group (*p* < 0.01). The RenalGuard group produced on average more hours of higher urine output more quickly that the standard group (*p* = 0.0003) (Figure 2).

### 3.3. Urine Output

Urine output in the first 24 hours was significantly higher in the RenalGuard group in comparison to the standard group (Table 2). There was no significant difference in the second 24 hours of the observation period.

### 3.4. Fluid Balance, Electrolyte Replacement, Loop Diuretic, and Sodium Bicarbonate Administration

Fluid balance at 48 hours was comparable in both groups as was deviation from clinician set daily fluid balance. Electrolyte replacement, dose of loop diuretics, and number of sodium bicarbonate administrations during the protocol period were also comparable (Table 3). Creatinine and blood urea nitrogen (BUN) trends can be found in Table 4. There were no significant differences in acid-base status, electrolytes, or Creatinine Phosphokinase levels at 48 hours between groups (data not shown).

### 3.5. Protocol Cessation

The RenalGuard protocol was stopped prematurely in two cases, one for haemodynamic instability and one for the initiation of renal replacement therapy, and both are included in the analysis. A third patient, who is also included in the analysis, had the RenalGuard protocol discontinued at 48 hours of treatment by the treating clinician due to concerns over excessive urine output (30 865 mL over 48 hours) even though the fluid balance had not deviated from the set target. In the remaining 5 patients the RenalGuard was discontinued in line with our rhabdomyolysis protocol for CK lower 5000 U/l after at least 48 hours of treatment in 4. A further case switched to the standard protocol after 72 hours. One patient had a brief interruption of the protocol to change a blocked urinary catheter but was still included in the final analysis. There were no episodes of hypotension requiring the institution of new vasopressors nor were there any episodes of pulmonary oedema requiring invasive or noninvasive ventilation.

## 4. Discussion

In critically ill patients with rhabdomyolysis, incorporating automatic fluid management using the RenalGuard device increased the number of hours with urine output >2 mL/kg/h in the first 48 hours of treatment. This was primarily due to a reduction in time taken to achieve target urine output compared to a standard protocol in the first 24 hours (Figure 2). These findings are supported by the observation that absolute urine output and urine output per kg per hour were only significantly higher in the RenalGuard group in the first 24 hours of therapy but not thereafter. However, overall, an average target urine output of 2 mL/kg per hour was reached in the RenalGuard group for the whole 48 hours but not in the control group. We hypothesise that automatic urine output replacement might be superior by eliminating the need for error-prone manual calculations and delayed infusion pump adjustments, by avoiding transient intravascular hypovolaemia.

Fluid balances at 48 hours as well as Creatinine trends were similar in both groups. Electrolyte replacement and the administration of loop diuretics and sodium bicarbonate did not significantly differ between the groups demonstrating safety and the efficacy of an automated fluid management approach.

### 4.1. Strengths and Limitations

This single centre service evaluation is with only a small number of patients aimed at demonstrating that we could use the RenalGuard to implement our current Rhabdomyolysis treatment protocol safely and effectively; these goals were achieved and we were able to easily employ this device to provide comparable management to our current standard of care. To our knowledge, this is the first application of the RenalGuard in critically ill patients with significant rhabdomyolysis. As this was not a trial, small sample size, lack of prospective randomisation, and the use of retrospectively collected control data mean that any findings are susceptible to bias and random effects; therefore, the results can only be taken as hypothesis generating only. In particular the mandatory presence and surveillance by an investigator of patients using RenalGuard during the initial stages of treatment may have enforced better compliance with treatment protocols. Finally, the study was too small to analyse any effect of the different protocols on kidney function and development of AKI, which is the overall intent of fluid administration in Rhabdomyolysis.

## 5. Conclusions and Future Research

We have demonstrated that the use of a protocol incorporating RenalGuard is effective at rapidly increasing urine output without the need for additional electrolyte replacement and diuretic administration and can achieve results at least comparable to manual fluid management in these patients. A randomised trial would be required to assess the effect of diuresis-matched hydration on prevention of acute kidney injury in severe rhabdomyolysis to demonstrate a therapeutic benefit of automated fluid management to these patients our preliminary data would support further study of the RenalGuard device in this context as well as in other groups of critically ill patients who might benefit from precise fluid management.

## Figures and Tables

**Table 1:** Baseline Characteristics (median, interquartile range) and number (percentage); ICU, Intensive Care Unit, IQR, interquartile range; CK, Creatine Phosphokinase; U/l, international units per Litre.

	Manual	RenalGuard
Number (*n*)	28	8
Age (median, IQR) (years)	31 (21.5–43)	44 (24.5–63)
Gender (*n*, %)		
Female	6 (21.4)	1 (12.5)
Male	22 (78.6)	7 (87.5)
BMI (median, IQR) (kg/m^2^)	24.9 (22.2–26.9)	25 (23.5–29.5)
Creatinine at ICU admission (median, IQR) (mmol/L)	102 (75–133)	99 (92–112)
Peak CK (median, IQR) (U/l)	14 431 (9372–21578)	13 965 (10606–33225)
Primary cause of rhabdomyolysis (*n*, %)		
Seizure	1 (3.6)	1 (12.5)
Trauma	21 (75.0)	5 (62.5)
Muscle/limb ischaemia	4 (14.3)	2 (25.0)
Postoperative	2 (7.1)	0 (0.0)

**Table 2:** Comparison of urine output between groups (median, interquartile range).

	Manual	RenalGuard	*p* value
*Absolute urine output (mL)*			
First 24 hours	3006 (2263–4188)	4054 (3682–7363)	0.01
Second 24 hours	4228 (3246–7655)	4311 (3173–11263)	0.58
Observation period (48 hours)	7228 (6044–9454)	7834 (7103–20259)	0.22
*Urine output (mL/kg/h)*			
First 24 hours	1.6 (1.2–2.2)	2.6 (1.7–4.4)	0.03
Second 24 hours	2.4 (1.9–2.8)	2.3 (1.6–6.7)	0.88
Observation period (48 hours)	1.9 (1.7–2.6)	2.3 (1.7–6.0)	0.41

**Table 3:** Fluid balance, electrolyte replacement, and furosemide and sodium bicarbonate administration (median, interquartile range). Deviation from fluid balance is a delta from set target so positive and negative changes do not cancel each other out.

	Manual	RenalGuard	*p* value
Actual fluid balance at 48 hours (mL)	2024 (178–4096)	2215 (1278–5170)	0.39
Deviation (above or below) from set target balance (mL)	1720 (−201–2595)	1253 (528–3545)	0.87

Magnesium (g)	0 (0–10)	5 (0–12.5)	0.79
Potassium (mmol)	40 (0–160)	0 (0–40)	0.26
Phosphate (mmol)	0 (0–40)	20 (0–50)	0.69
Sodium bicarbonate 1.26% (*n*, %)	13 (46.4)	4 (50.0)	0.93
Furosemide (mg)	98 (0–110)	60 (30–102)	0.67

**Table 4:** Creatinine and blood urea nitrogen (BUN) trends in mmol/L, respectively, median (interquartile range).

Patient number	BUN (mmol/L)			Creatinine (mmol/L)		
	First	Maximal	Last	First	Maximal	Last
1	13	24	6	92	232	55
2	6.3	5.3	4.9	121	163	25
3	3.5	9.8	5	149	149	95
4	2.2	5.6	5.6	102	102	49
5	5.7	4.8	4.8	107	107	69
6	4.8	11.2	7.2	77	116	88
7	8.4	16.1	15.8	88	388	188
8	5.4	5.4	3.7	96	96	82

RenalGuard	6 (5–8)	8 (5–12)	5 (5-6)	99 (92–112)	115 (103–160)	66 (56–87)

Manual				102 (75–133)	113 (86–156)	57 (48–67)
